# Determinants of Stunting at 6 Weeks in the Northern Cape Province, South Africa

**DOI:** 10.3389/fpubh.2020.00166

**Published:** 2020-06-05

**Authors:** Maretha le Roux, Mariette Nel, Corinna Walsh

**Affiliations:** ^1^Department of Nutrition and Dietetics, University of the Free State, Bloemfontein, South Africa; ^2^Department of Biostatistics, University of the Free State, Bloemfontein, South Africa

**Keywords:** growth, malnutrition, stunting, double-burden, South Africa

## Abstract

The first 1,000 days from conception to 24 months is a critical period for healthy growth and development. In South Africa, stunting (weight-for-length below −2SD from the WHO reference mean) is a major public health issue with significant health consequences. We determined associations between demographic, health, and anthropometric indicators of mothers and their infants. A cross-sectional study was conducted in the Northern Cape. All mothers with 5- to 7-week-old babies visiting PHC facilities were invited to participate. A questionnaire was completed in a structured interview with each mother. Age and length of the baby at 6 weeks were used to determine stunting, while the weight and height of the mother were measured for body mass index (BMI). Eight hundred questionnaires were completed in 92 facilities. The median age of mothers was 26 years (IQR 20–30 years) and 44.9% were married. Only 40.1% had completed school or tertiary education and almost 40% relied on a government grant as the main source of income. Two-thirds (64.9%) had not planned the pregnancy and 17% were on antiretroviral therapy (ART). More than a quarter (26.1%) smoked cigarettes or used snuff during pregnancy, while 9.4% drank alcohol. At 6 weeks, 31% of boys and 14% of girls had a length-for-age below the WHO reference values, while 25.4% of mothers were classified as obese and 24.6% as overweight. More than 70% had a waist circumference above 80 cm. Significantly more mothers with stunted babies weighed less [−6 kg; −1 kg] and were shorter [−4 cm; −1 cm] than mothers with babies who were not stunted. Compared to babies who were not stunted, significantly more babies of mothers who lived in informal housing [−19.7%; −3.2%], relied on a grant [−19.7%; −3.2%], smoked/ snuffed [7.6%; 23.5%], and used alcohol during pregnancy [0.3%; 11.5%] were stunted. The following factors significantly increased the risk of having a stunted baby at 6 weeks: living in informal housing vs. formal housing (RR: 0.68, 95% CI [0.5; 0.9]); smoking or using snuff during pregnancy (RR: 1.74, 95% CI [1.3; 2.3]); using alcohol during pregnancy (RR: 1.5, 95% CI [1.1; 2.2]); both smoking and using alcohol during pregnancy (RR: 1.97, 95% CI [1.4; 2.9]). We confirmed the coexistence of under- and over-nutrition among mothers and their babies, possibly indicating that stunting in childhood may predispose to overweight and obesity in adulthood in a vicious cycle that affects generation after generation. Interventions aimed at poverty alleviation and encouraging healthy lifestyles with an emphasis on healthy eating, smoking cessation and abstaining from alcohol before pregnancy are urgently required.

## Introduction

Exposures and experiences in a child's early environment—in the womb and early life—can affect survival and have long-lasting effects throughout life, in terms of health, development, and well-being. The first 1,000 days of life, ranging from conception to 24 months, is a critical period for growth and development (1). Children's growth and development are influenced by the environment, nutrition, maternal health, and parental care. These factors play an important role in a child reaching her/his full potential (2, 3). Good nutrition, particularly in early childhood, is critical to the positive health outcomes of adults (2–4), and children's nutritional status can be viewed as a good indicator of a community's state of health (5).

Childhood stunting (defined as a length-for-age below −2SD from the reference WHO mean) often begins before birth and continues to be a public health issue in many African countries (6). Africa is currently the only continent where stunting rates continue to rise (7), with 29% of African children classified as stunted in 2018. A systematic review by Said-Mohamed et al. (8) indicates that despite economic transition over the past 40 years in South Africa, stunting persists at a significant level.

Early malnutrition and growth failure, specifically stunting, are associated with increased mortality, reduced human capital, increased risk of chronic disease, and long-term intergenerational consequences (1, 4, 9, 10). Stunting is associated with an increased risk of mortality from infectious diseases in childhood. It leads to irreversible physical and cognitive damage and poorer educational outcomes later in childhood and adolescence, with economic consequences for the individual, household, and community. Stunted children who experience rapid weight gain after 2 years of age have an increased risk of becoming overweight or obese later in life, with an associated higher risk of noncommunicable diseases (11–13). Childhood stunting costs low- and middle-income countries billions of dollars in future revenue losses through reduced economic productivity, particularly through lower wages, lower physical and mental capabilities, and more days away from work as a result of illness (14).

According to the South African National Health and Nutrition Examination Survey (SANHANES-1) of 2012, boys living in the Northern Cape (NC) had a 22.8% stunting and 8.7% severe stunting prevalence and girls a 15% stunting and 3.9% severe stunting prevalence. This is among the highest in the country (15). Other studies undertaken in the NC have confirmed high stunting levels in certain communities—40% amongst preschool children in the Hantam subdistrict (16) and 26% in the Frances Baard district among HIV positive mother–child pairs (17).

Thus, the problem of stunting begins at an early age and has long-term consequences for both the individual and society as a whole (18). However, stunting often goes unrecognized, especially in communities where short stature is so common that it seems normal (19, 20). Data related to the prevalence of stunting at birth and during early infancy in the NC are lacking. Given the impact of stunting on health and development, such information is required for the formulation of policies and programs to address stunting and its consequences. The objective of this study was thus to determine how stunting is associated with sociodemographic, health, and anthropometric status of mothers and their 6-week-old infants to determine where interventions to address stunting should be focused.

## Methods

### Study Design, Setting, and Participants

A cross-sectional study was conducted in all districts in the Northern Cape Province of South Africa during 2017. Inclusion criteria included all mothers with 5- to 7-week-old babies who attended any of the 92 PHC facilities in the five districts in the NC and signed written informed consent on the day of the visit. Mothers with mental disabilities were excluded from the study.

### Techniques and Procedures

Approval was obtained from the Health Sciences Research Ethics Committee of the University of the Free State (ECUFS-HSD2017/0181) as well as the Provincial Health Research and Ethics committee of the Department of Health in the NC (NC-2017RP55-938).

The questionnaire (Supplementary Material) was developed by the research team based on an in-depth literature review in which variables that had been assessed in relevant studies and that were directly related to the aims and objectives of the current study were identified. The order in which questions were asked was carefully considered to avoid influencing the participant's answers to subsequent questions, and the time required to complete the questionnaire was kept as short as possible to avoid participant fatigue (each interview took 20–30 min to complete). The questionnaire was completed by qualified dietitians and nutritionists in a structured interview with each mother during their 6-week visit to the health facility, ensuring that both literate and illiterate participants could be included. Information about sociodemographic conditions, pregnancy, birth, care practices, and infant feeding practices was collected.

The anthropometric information of the baby at birth was obtained from the Road to Health booklet, while the anthropometry of the baby and mother at 6 weeks was measured by the researchers. Weight and recumbent length of the baby at birth and 6 weeks were measured to determine stunting, while the weight and the height of the mother were measured for body mass index (BMI). At 6 weeks, all anthropometric measurements were taken by trained dietitians and nutritionists by following measurement protocols recommended by the World Health Organization for children (21) and by the International Standards for Anthropometric Assessment for adults (22), using regularly calibrated measuring equipment.

Those participants who were identified as requiring health and nutrition education or additional medical management were referred to appropriate healthcare services.

### Data Analysis

Data were analyzed by the Department of Biostatistics at the University of the Free State using SAS software (copyright, SAS Institute Inc.; SAS and all other SAS Institute Inc. product or service names are registered trademarks or trademarks of SAS Institute Inc., Cary, NC, USA). Descriptive statistics, namely frequencies and percentages for categorical data and medians and ranges for numerical data, were calculated. For associations between variables, descriptive statistics for the median or percentage difference were calculated and described using 95% confidence intervals for the median or percentage difference between the group of mothers with a stunted child and the group without a stunted child at 6 weeks. The relative risk (RR) to develop stunting at 6 weeks was described using 95% confidence intervals for the RR. If a participant was unsure of a particular response to a question, this was indicated as missing, hence the difference in numbers who answered.

## Results

A total of 800 mothers were interviewed (44.0% from Frances Baard, 20.5% from JT Gaetsewe, 5.9% from Namakwa, 10.8% from Pixley Ka Seme, and 18.8% from ZF Mgcawu).

### Sociodemographic Status

Results related to the sociodemographic status of the sample are depicted in Table 1, while associations between sociodemographic variables and the occurrence of stunting are depicted in Table 2.

**Table 1:** Sociodemographic information.

	***n***	**%**
**Age of mother (*****n*** **=** 799*)		
<19 years	82	10.3
19–35 years	640	80.1
>35 years	77	9.6
**Marital status (*****n*** **=** **800)**		
Married	359	44.9
**Home language (*****n*** **=** **800)**		
Afrikaans	416	52
English	20	2.5
Setswana	305	38.1
IsiXhosa	31	3.9
Other African languages	16	1.9
Foreign languages	12	1.7
**Level of education (*****n*** **=** 797*)		
Grade 1–7	79	9.9
Grade 8–11	379	57.5
Grade 12	229	28.7
Still at school	19	2.4
Tertiary	91	11.4
**Main source of income (*****n*** **=** 799*)		
Permanent salary	355	44.4
Temporary/contract/piece job	94	11.8
Grant/pension	312	39.1
Support from family	38	4.8
**Type of water in the household (*****n*** **=** 799*)		
Piped water within the dwelling	362	45.3
Piped water within the stand	255	31.9
Piped water within 200 m from the stand	147	18.4
Piped water more than 200 m from the stand	34	4.3
No access to piped water	1	0.1
**Type of house (*****n*** **=** 799*)		
Formal house or flat	393	49.2
Reconstruction and Development Programme (RDP) house	238	29.8
Shanty or tin	165	20.7
Wendy house or mud house	3	0.4
**Toilet facilities (*****n*** **=** **800)**		
Flush toilet in the house	364	45.5
Flush toilet outside the house	362	45.3
Bucket	33	4.1
Hole or veld	12	1.5
Pit toilet	27	3.4
Sakkies (used in Platfontein by the Bushmen)	2	0.3
**In the past 4 weeks, no food to eat of any kind in the house because of a lack of resources to obtain food (*****n*** **=** **800)**	180	22.5

* *If a participant was unsure of a particular response to a question, this was indicated as missing, hence the difference in numbers who answered*.

**Table 2:** Associations between sociodemographic information of mothers with babies who were stunted and mothers with babies who were not stunted at 6 weeks.

**Variables**	**Stunted**	**Not stunted**	**95% CI for med or % diff**	**Relative risk (RR)**	**95% CI for RR**
Median age of mother at 6 weeks (IQR)	27 (22–32)	26 (21–31)	0; 2		
Married (%)	45.3	44.8	−7.7; 9.0		
Afrikaans speaking (%)	58.2	50.3	−0.5; 16.1	1.35	1.0; 1.8
Median level of education (IQR)	10 (9–12)	11 (10–12)	−1; 0		
Permanent salary as main source of income (%)	38.8	46.0	−15.1; 1.3	0.79	0.6; 1.0
Piped water in house (%)	42.0	46.2	−12.4; 4.3	0.91	0.7; 1.2
Formal housing, not RDP (%)	40.0	51.7	−19.7; −3.2*	0.68	0.5; 0.9*
Toilet inside house (%)	42.9	46.2	−11.4; 5.2	0.9	0.7; 1.3
Dependent on a grant (%)	51.7	40.0	−19.7; −3.2*	1.2	1.0; 1.6
Food insecure	25.9	21.6	−2.6; 12.0	1.2	0.9; 1.6
Mother younger than 19 years (%)	10.0	10.3	−4.8; 5.5	0.97	0.6; 1.5

* *Statistically significant difference*.

The median age of mothers was 26 years (range 10–46 years; interquartile range 20–30 years). The age categories were chosen to facilitate comparisons with the most recent South African Demographic and Health Survey (23). The majority of mothers were between 19 and 35 years of age (80.1%). Ten percent were younger than 19 years and 10% older than 35 years. Almost 10% (9.9%) of mothers had primary school education and only 28.7% had completed matric, while 11.4% had completed a tertiary qualification. Less than half of the mothers (44.9%) were married. Fifty-two percent (52%) of mothers spoke Afrikaans at home and nearly 40% (38.1%) Setswana. Almost 40% of participants relied on a government grant as their main source of income.

Nearly 80% (79%) of participants lived in a brick-built house, 77.2% had piped water either within the dwelling or on the stand (outside the house), and 90.8% had a toilet either in the house or on the stand. Although 21% were staying in informal housing, only 4.4% had either no access to piped water or more than 200 m from the stand, and 9.3% were making use of a toilet other than a flush toilet. Almost a quarter (22.5%) of participants reported that they were food insecure.

Significantly fewer mothers with stunted babies lived in a brick house (not RDP) (40.0%) compared to mothers with babies who were not stunted (51.7%) (95% CI for percentage difference −19.7%; −3.2%). When considering RRs, living in informal housing vs. formal housing significantly increased the risk of having a stunted baby at 6 weeks: (RR: 0.68, 95% CI [0.5; 0.9]). Also, significantly more mothers with stunted babies were dependent on a grant (51.7%) compared to mothers with babies who were not stunted (40.0%); 95% CI for the percentage difference [−19.7%; −3.2%]. Although differences did not reach significance, a smaller percentage of mothers who had a fixed salary as their main source of income had a stunted baby (38.8%), compared to mothers who did not have a fixed salary as income (46.0%), 95%CI [−15.1%; 1.3%], RR: 0.79. The same was true for the prevalence of food insecurity (answered “yes” to the question “In the past 4 weeks, was there ever no food to eat of any kind in your house because of lack of resources to obtain food?”), 95% CI [−2.6%; 12.0%], RR:1.22.

### Pregnancy-Related Information

Information related to the pregnancy is shown in Table 3, while associations between pregnancy-related information and the occurrence of stunting are depicted in Table 4.

**Table 3:** Pregnancy-related information.

	***N***	**%**
Used family planning (*n* = 800)	271	33.9
**Family planning method used (*****n*** **=** **269)**		
• Tablets	43	15.0
• Injection	205	76.2
• Condom	6	2.2
• Implanon	11	4.1
• Sterilized	3	1.1
• IUD	1	0.4
• Pregnancy planned (*n* = 797)	280	35.1
• Attended an antenatal clinic (*n* = 800)	777	97.1
**Weeks pregnant when visited clinic for the first time (*****n*** **=** **755)**		
• 1–10	298	39.5
• 11–20	348	46.1
• 21–30	90	11.9
• 31+	19	2.5
• Median number of antenatal care visits (*n* = 758)	6	
**Medication and/or supplements used during pregnancy (*****n*** **=** **777)**		
• Iron	694	89.3
• Folic acid	667	85.8
• Calcium gluconate	603	77.6
• Micronutrients	581	74.8
• Iron, folic acid, calcium gluconate, and micronutrients	419	54.0
• Enriched porridge or other nutrition supplements	68	8.8
• ART	132	17.0
• Hypertension	50	6.4
• Diabetes	11	1.4
• Other	7	0.9
• If on ART treatment, knew status before falling pregnant (*n* = 130)	92	70.8
• Received breastfeeding education during antenatal care visits (*n* = 774)	659	85.1
**If yes, from: (*****n*** **=** **659)**		
• Professional nurse	316	48.0
• Dietitian	46	7.0
• Community health worker (CHW)	207	31.4
• Do not know who	4	0.6
• Private doctor	4	0.6
• Nurse and dietitian	28	4.3
• Nurse and CHW	53	8.0
• Doctor and CHW	1	0.2
• Child registered for a child support grant (*n* = 798)	266	33.3
• Smoked or snuffed during pregnancy (*n* = 800)	209	26.1
• Used recreational drugs during pregnancy (*n* = 800)	3	0.3
**Used alcohol during pregnancy (*****n*** **=** **799)**		
• Yes	75	9.4
• Yes, before I knew I was pregnant	117	14.6

**Table 4:** Associations between pregnancy-related information of mothers with babies who were stunted and mothers with babies who were not stunted at 6 weeks.

**Variables**	**Stunted**	**Not stunted**	**95% CI for % or med diff**	**Relative Risk (RR)**	**95% CI for RR**
**Pregnancy information**					
Attended antenatal clinic (%)	96.5	97.3	−4.9; 1.6	0.79	0.4; 1.6
Weeks pregnant at first clinic visit (med and IQR)	12 (8–18)	12 (8–20)	−1; 1		
Number of visits to clinic during pregnancy (med and IQR)	6 (4–7)	6 (4–7)	0; 0		
Gestational age (med weeks and IQR)	38 (36–40)	39 (38–40)	−1; 0		
Exclusive breastfeeding during past 24 h (%)	79.4	78.4	−6.4; 7.4	1.0	0.7; 1.5
**Supplements received during pregnancy**					
Iron (%)	90	86.9	−2.9; 7.7	1.24	0.8; 1.9
Folic acid (%)	83.5	84.4	−7.7; 4.8	0.91	0.6; 1.3
Calcium gluconate (%)	71.8	77	−13.1; 1.9	0.80	0.6; 1.1
Micronutrients (%)	75.3	73.2	−5.6; 9.0	1.01	0.7; 1.4
Enriched porridge (%)	10.0	8.3	−2.6; 7.5	1.12	0.7; 1.7
**Medication used during pregnancy**					
ART (%)	19.4	16.2	−2.9; 10.3	1.19	0.9; 1.7
Hypertension (%)	9.4	5.4	−0.1; 9.6	1.52	1.0; 2.3
Diabetes (%)	0.6	1.6	−2.4; 1.8	0.40	0.1; 2.6
**Lifestyle**					
Smoked or snuffed during pregnancy (%)	38.2	22.9	7.6; 23.5*	1.74	1.3; 2.3*
Used alcohol during pregnancy (%)	13.5	8.3	0.3; 11.5*	1.52	1.1; 2.2*
Both smoked and used alcohol during pregnancy	11.2	4.8	2.0; 12.2*	1.97	1.4; 2.9*
No use of illicit drugs (%)	99.4	99.7	−2.9; 0.7	0.68	0.1; 3.4
Use amphetamines during pregnancy (Tik) (%)	0.0	0.16	−0.9; 2.1	1.29	1.2; 1.3
Use heroin during pregnancy (%)	0.6	0.0	−0.2; 3.3	4.43	3.8; 5.1*
Use cannabis during pregnancy (%)	0.0	0.16	−0.9; 2.1	1.29	1.2; 1.3

* *Statistically significant difference*.

In terms of pregnancy-related questions, only one-third (33.9%) of the participants indicated that they had used family planning. Injectable contraceptive (76.2%) was the most popular method, followed by oral contraceptives (16%). Nearly two-thirds (64.9%) of the participants did not plan the pregnancy. Almost all participants (97.1%) attended antenatal care clinics, and 85.6% did so before 20 weeks of pregnancy and more than half (54%) had six or more visits.

Although the supplementation coverage per supplement was high, only 54% of participants received all supplements—iron, folic acid, calcium gluconate, as well as micronutrients as prescribed by Basic Antenatal Care (BANC) guidelines. Seventeen percent (17%) of participants were on antiretroviral therapy (ART) and 71% knew their HIV status before this pregnancy. Breastfeeding education was given to the majority of the participants (85.1%), mainly by professional nurses (48%), followed by Community Health Care workers (31.4%). A third of the participants (33.3%) had already registered their baby for a child support grant. More than a quarter of the participants smoked cigarettes or snuffed during their pregnancy, while 9.4% reported drinking alcohol and another 14.6% reported drinking alcohol before they knew they were pregnant.

Although more mothers with stunted babies were on ART (19.4%) than mothers with babies who were not stunted (16.2%), the 95% CI percentage difference was not significant [−2.9%; 10.3%]; RR 1.19, 95% CI for RR [0.9; 1.7]. The same was true of mothers who used medication for hypertension [−0.1%; 9.6%], RR 1.5, 95% CI for RR [1.0; 2.3].

Significantly more mothers with stunted babies smoked or snuffed (38.2%) than mothers with babies who were not stunted (22.9%), 95% CI for the percentage difference of [7.6%; 23.5%]. Similarly, significantly more mothers with stunted babies used alcohol during their pregnancy (13.5%) than mothers with babies who were not stunted (8.3%), 95% CI for the percentage difference of [0.3%; 11.5%]. The same was seen in terms of mothers who both smoked and used alcohol during pregnancy (11.2% had stunted babies compared to 4.8% who did not have a stunted baby, 95% CI [2.0; 12.2]). The risk of having a stunted baby at 6 weeks was significantly increased in mothers who were smoking or using snuff during pregnancy (RR: 1.74, 95% CI [1.3; 2.3]); using alcohol during pregnancy (RR: 1.5, 95% CI [1.1; 2.2]); and both smoking and using alcohol during pregnancy (RR: 1.97, 95% CI [1.4; 2.9]). Although the number of women who used recreational drugs during pregnancy was small, this also significantly increased the risk of having a stunted baby (RR of 1.29 for amphetamines, 4.43 for heroin and 1.29 for cannabis).

### Infant Feeding-Related Information

At 6 weeks, the majority of mothers (79%) exclusively breastfed their babies, while 14% mix fed (MF) and 7% exclusively formula-fed (EFF) their babies. Reported reasons for not breastfeeding exclusively included the following: cracked nipples or mastitis, the baby was constipated, or a belief that the baby was not getting enough milk. Although the majority of the mothers who MF gave formula, nearly 20% gave either cow's milk, muthi (traditional medicines), or solid foods (Table 5).

**Table 5:** Infant feeding information.

	***n***	**%**
**Baby ate or drank during the past 24 h: (*****n*** **=** **800)**		
• Exclusive breastfeeding (EBF)	629	78.6
• Mixed feeding (MF)	114	14.3
• Exclusive formula feeding (EFF)	57	7.1
**If EFF, reason: (*****n*** **=** **57)**		
• Breastfeeding-related problems	40	70.2
• HIV positive	4	7.0
• Medical condition	3	5.3
• Other	10	17.5
**If MF, gave: (*****n*** **=** **114)**		
• Formula	79	69.3
• Water, including sugar water	41	34.2
• Solid foods	2	1.8
• Other (cow's milk, muthi)	19	16.7
**If MF, reason: (*****n*** **=** **110)**		
• Breastfeeding-related problems	46	41.8
• Went back to school or work	22	20.0
• To give gripe water for cramps	15	13.6
• Cultural beliefs	18	16.4
• Other	9	8.2

### Anthropometric Information

The anthropometric results for the babies at birth and 6 weeks are presented in Table 6, while those of mothers at 6 weeks are depicted in Table 7.

**Table 6:** Anthropometry of babies at birth and 6 weeks.

	**% Male at birth (*n* = 388)**	**% Male at 6 weeks (*n* = 387)**	**% Female at birth (*n* = 367)**	**% Female at 6 weeks (*n* = 366)**
**Weight for age (*****n*** **=** **749)**				
Severe underweight	3.6	7.7	2.8	3.8
Moderate underweight	9.4	11.4	7.4	3.3
Normal weight	86.8	80.6	88.7	91.3
Overweight	0.3	0.3	1.1	1.6
**Length for age (*****n*** **=** **741)**				
Severe stunting	8.7	13.8	5.8	4.7
Moderate stunting	9.0	17.0	7.2	9.4
Normal	76.3	67.6	70.2	80.0
Tall	6.1	1.6	16.9	6.1
**Weight for height**				
Severe wasting	13.6	4.7	11.2	3.6
Moderate wasting	14.2	7.1	16.7	5.0
Normal	68.5	77.5	68.2	80.3
Overweight	3.8	10.7	3.9	11.1
**Head circumference**				
Severe	3.7	3.1	2.2	1.7
Moderate	5.0	4.7	4.4	1.7
Normal	89.5	88.6	87.0	71.8
Above normal	1.9	3.6	6.4	24.8

**Table 7:** Anthropometry of mothers at 6 weeks.

	***N***	**%**
**BMI (*****n*** **=** **790)**		
<18.5 kg/m^2^ (Underweight)	47	5.9
18.5–24.9 kg/m^2^ (Normal)	346	43.6
≥25–29.9 kg/m^2^ (Overweight)	195	24.6
≥30–39.9 kg/m^2^ (Obese)	175	22.0
≥40 kg/m^2^ (Morbidly obese)	27	3.4
**MUAC (*****n*** **=** **798)**		
<23 cm (Underweight)	87	10.9
≥23– <30 cm (Normal)	450	56.4
≥30 cm (Overweight)	191	23.9
**Waist circumference (*****n*** **=** **786)**		
<80 cm	224	28.5
≥80 cm	562	71.5

At birth, the majority of babies had a weight-for-age in the normal category (boys—86.8%; girls—88.7%), and this remained more or less the same at 6 weeks. Unexpectedly, there were fewer tall children at 6 weeks than at birth. After investigating possible reasons for this, it was found that the method used by midwives and hospital staff to determine the length at birth was not always in accordance with WHO guidelines, while at 6 weeks when the correct method was used by the dietitians and nutritionists, 31% of boys and 14% of girls were found to be stunted. The majority of babies had a head circumference in the normal range (males—89.5%; females—87%).

In terms of the mothers, 25% were classified as overweight and a further 25% as obese. Only 6% of participants were underweight. At 6 weeks, more than 70% of mothers had a waist circumference above 80 cm.

The median weight of mothers with babies who were not stunted was 62 kg, which was significantly higher than the median weight of 60 kg in mothers with babies who were stunted (95% CI for the median difference [−6 kg; −1 kg]). The median height of mothers with stunted babies was 154 cm, which was significantly shorter than the median height of 157 cm in mothers with babies who were not stunted (95% CI for the median difference [−4 cm; −1 cm] Table 8).

**Table 8:** Associations between anthropometric variables of mothers with babies who were stunted and mothers with babies who were not stunted at 6 weeks.

**Variables**	**Stunted median (IQR)**	**Not stunted median (IQR)**	**95% CI for med diff**
Mother's weight at 6 weeks (kg)	60 (49–74)	62 (53–74)	−6; −1*
Mothers height at 6 weeks (cm)	154 (151–160)	157 (153–161)	−4; −1*
Mother's MUAC at 6 weeks (cm)	27 (24–31)	27 (24–30)	−1; 1
Mother's waist circumference at 6 weeks (cm)	87 (76–95)	87 (79–98)	−4; 1

* *Statistically significant difference*.

## Discussion

The large and representative sample size of the current study is a strength. We acknowledge that the findings are specific to the Northern Cape and may not reflect the situation in other provinces. The addition of biochemical measures of nutritional status would have added value, but were not possible due to the high cost. The most significant limitation is the fact that, in some cases, the birth length noted in the Road to Health booklet was not measured according to WHO recommendations, which meant that when some of the babies were measured correctly by dietitians at 6 weeks, they were shorter than at birth. For this reason, associations between the prevalence of stunting and other variables were not determined at birth, but only at 6 weeks.

Ten percent of mothers were younger than 19 years, with the youngest outlier being only 10 years old. The teenage pregnancy rate in the current study was similar to that reported in the most recent SADHS (23) where the Northern Cape had the second highest teenage pregnancy rate in the country (23). In the current study, 17.1% of babies who were stunted at 6 weeks were born to mothers <19 years old compared to the 71% born to mothers between 19 and 35 years. Young and Martorell (1) note that teenage girls are not fully developed and are in most cases still growing. Because of this, their nutritional requirements are higher than those of older mothers, increasing their risk of having a stunted baby. In the current study, however, the RR for mothers younger than 19 years to have a stunted baby was 0.97, 95% CI for RR [0.6; 1.5], indicating no difference in the risk of stunting between younger and older mothers in this population. The fact that several health indicators were compromised in all women, independent of age, may have contributed to this finding.

The results of the current study confirmed high levels of poverty in the study population. This is evidenced by the large percentage of mothers who had not completed high school (almost 70%) and almost 40% who relied on a government grant as their main source of income. A third of the participants had already registered their 6-week-old baby for a child support grant. Two in 10 (21%) mothers were living in informal housing, more than 50% did not have access to piped water in their dwelling, and about 1 in 10 households did not have access to a flush toilet. Almost a quarter (22.5%) of participants reported that they were food insecure. Similarly, the SANHANES-1 reported that in 2012, 22.8% of the participants from the Northern Cape were at risk of food insecurity and 20.7% were food insecure or hungry. According to the National Food Consumption Survey, underweight and stunting were more likely to be present in children from households in the Northern Cape who were at risk of hunger or experiencing hunger (24).

Studies from South Asian countries have reported that children from the poorest households are more than twice as likely to be stunted compared with children in the wealthiest households (25). Associations between variables in the current study showed that a significantly larger percentage of mothers with stunted babies lived in informal housing (95% CI [−19.7%; −3.2%]) and were dependent on a grant as their main source of income (95% CI [−19.7%; −3.2%]). Living in informal housing vs. formal housing significantly increased the risk of having a stunted baby at 6 weeks: (RR: 0.68, 95% CI [0.5; 0.9]).

In terms of health services, we identified inadequate uptake of family planning services and inadequate nutritional supplementation practices. Nearly two-thirds (64.9%) of participants did not plan the pregnancy. Almost all pregnant women attended antenatal clinics and the majority did so before the 20th week of pregnancy. Despite this, almost half did not receive the full set of supplements recommended by BANC guidelines and fewer than 10% (8.8%) were supplemented with enriched porridge according to the South African Integrated Nutrition Programme policy guidelines.

Only 71% of participants knew their HIV status before this pregnancy. At the time of data collection, 17% of participants were on ART. Although the difference did not reach statistical significance, more mothers with stunted babies were on ART (19.4%) than mothers with babies who were not stunted (16.2%), (95%CI [−2.9%; 10.3%]).

Due to the harmful effects on unborn children, smoking and alcohol consumption are both discouraged during pregnancy. Despite this, more than a quarter of the participants smoked cigarettes or snuffed during their pregnancy (26.1%). These findings are confirmed by those of the SANHANES-1 that reported high rates of smoking among women in the Northern Cape (15).

One in 10 mothers (9.4%) reported drinking alcohol during their pregnancy, while another 14.6% reported drinking alcohol before they knew they were pregnant. The alcohol consumption of mothers during pregnancy concurs with that observed in a previous study undertaken in the province by Fetal Alcohol Related Research (FARR). This study found that the prevalence of Fetal Alcohol Syndrome was several times higher in the Northern Cape than elsewhere in the world (26). Maternal alcohol consumption does not only impact on brain development but also increases the risk of growth retardation (27, 28).

When associations between stunting with smoking and alcohol consumption were determined, significantly more mothers with stunted babies smoked (95% CI [7.6%; 23.5%]), used alcohol during their pregnancy (95% CI [0.3%; 11.5%]), and both smoked and used alcohol (95% CI [2.0; 12.2]) than mothers with babies who were not stunted. A recent study in an impoverished community in the Northern Cape reported that birth weight was lower in the children of mothers who smoked or used alcohol during pregnancy, but was lowest in children whose mothers both smoked and used alcohol, showing a cumulative effect of combined exposure (29). This was confirmed by the significantly increased RR to have a stunted baby of 1.74 for smoking and snuffing, 1.52 for using alcohol, and 1.97 for both smoking and using alcohol during pregnancy in the current study.

Nearly 80% (78.6%) of mothers reported exclusively breastfeeding their babies at 6 weeks. This is similar to the rates of exclusive breastfeeding reported in the South African Prevention of Mother-to-Child Transmission (SAPMTCT) Evaluation study of 2012–2013 where exclusive breastfeeding rates of 76% between 4 and 8 weeks were reported in the Northern Cape (30). The relatively high rates of exclusive breastfeeding could be attributed to the financial benefit of breastfeeding instead of formula feeding as well as the fact that 85% reported receiving breastfeeding counseling during antenatal visits. Most of the breastfeeding counseling was provided by professional nurses and Community Health Workers who are trained by dietitians. Despite this, breastfeeding-related challenges were still the most common reason why mothers who were not exclusively breastfeeding had started mixed feeding or formula feeding. The high prevalence of stunting in 6-week-old babies who were reportedly exclusively breastfed was unexpected, but may be related to the general poor nutritional and health status of mothers.

At 6 weeks, 31% of boys and 14% of girls were classified as stunted, while 25% of mothers were classified as overweight and a further 25% as obese and more than 70% had a waist circumference above 80 cm. Furthermore, a significantly higher percentage of mothers with stunted babies were lighter (95% CI [−6 kg; −1 kg]) and shorter (95% CI [−4 cm; −1 cm]) than mothers with babies who were not stunted. These results show that mothers with stunted babies were more likely to have been stunted as children themselves, thus never reaching their full height potential and predisposing them to an increased risk of becoming overweight as adults (31). Even though mothers with stunted babies were lighter than those with babies who were not stunted, a large percentage had a BMI in the overweight and obese category. The occurrence of undernutrition in children together with the high prevalence of overweight in adults confirmed a double burden of malnutrition in this population. South Africa has experienced rapid urbanization that has resulted in a nutrition transition, characterized by a shift from healthier traditional diets to a more Western unhealthy diet and sedentary lifestyle associated with lower energy expenditure, also contributing to overweight and obesity (32).

## Conclusion and Recommendations

In this study, we confirmed the coexistence of under- and overnutrition among mothers and their babies, possibly indicating that stunting in childhood predisposes to overweight and obesity in adulthood in a vicious cycle that affects generation after generation. To address stunting, interventions aimed at poverty alleviation and encouraging healthy lifestyles with an emphasis on maintaining a healthy weight, healthy eating, smoking cessation, and abstaining from alcohol before pregnancy are urgently required. These should be culturally relevant and focus on addressing both determinants of stunting as well as barriers that prevent women from following a healthy lifestyle during pregnancy. Furthermore, there is a need to strengthen adolescent and youth health services to combat the high teenage pregnancy rate and to improve linkages with disciplines such as agriculture that can assist in the fight against food insecurity. Finally, adequate training of healthcare workers who assess the length of newborn babies should ensure that reliable measures are taken at birth.

## Data Availability Statement

The raw data supporting the conclusions of this article will be made available by the authors, without undue reservation to any qualified researchers.

## Ethics Statement

The study involved human participants and was reviewed and approved by the Health Sciences Research Ethics Committee, University of the Free State. The participants provided their written informed consent to participate in this study.

## Author Contributions

MR wrote the protocol, supervised the fieldwork, and wrote the article. MN assisted with the planning of the study and statistical analysis of the data. CW supervised MR, and assisted with the protocol, interpretation of data, and writing of the article.

## Conflict of Interest

The authors declare that the research was conducted in the absence of any commercial or financial relationships that could be construed as a potential conflict of interest.
