# Supplementary material for: Determinants of Stunting at 6 Weeks in the Northern Cape Province, South Africa
Source: Front Public Health. 2020 Jun 5;8:166. doi: 10.3389/fpubh.2020.00166 (PMC7289919; doi:10.3389/fpubh.2020.00166)
Supplement: Supplementary file 1 [file Data_Sheet_1.PDF]

STUNTING AT BIRTH QUESTIONNAIRE

Only interview mothers:	That have signed consent<br>Delivered a live baby in past 6 weeks	**For Office use**

Instructions - Circle the appropriate number or write your answer in the space provided. — 1-3 Interview number

1.1 Name of facility ……………………………………………………………………………. — 4-5

1.2 Name of facility where your baby was born ……………………………………………….. — 6-7

1.3 Date questionnaire is completed ……… / ………. / ……….. (dd/mm/yy) — 8-13 (d d m m y y)

PART I: RESPONDENT PROFILE

Demographic information

2.1 How old are you in years?

…………… — 14-15

2.2 On which date was your baby born? — 16-21 (d d m m y y)

……………………………………..

2.3 What was the gestational age of the baby? (See RtHB p 6) — 22

……………………….. Weeks

2.4 Are you married? — 23

- Yes
- No

2.5 What is your home language? — 24

- 1 Afrikaans
- 2 English
- 4 SeTswana
- 5 IsiXhosa
- 7 Other, specify…………………………………….. — 25-26

2.6 What is your highest level of education?

…………………………………………….

2.7 What is the main source of income in the household?

…………………………… — 30-31

2.8 What kind of water does your household have access to? — 32

- Piped water within the dwelling
- Piped water within the stand
- Piped water within 200m from stand
- Piped water more than 200m from stand
- No access to piped water

2.9 What type of house are you staying in? — 33

- Formal house
- RDP house
- Shanty/Tin

☐ Other, please specify……………………………….. | 34-35

2.10 What toilet facilities are available in the house?

☐ Toilet in the house | 36
☐ Toilet outside the house
☐ Bucket
☐ Other, please specify ………………………………. | 37-38

2.11 How many children has the mother had, that are alive, including this child? (See RtHB p 4)

……………………………. | 39

2.12 If more than one child, how old are the others?

……………… | 40-41
……………… | 42-43
………………. | 44-45
……………… | 46-47
………………. | 48-49
………………. | 50-51

Pregnancy related questions

3.1 Did you use family planning?

☐ Yes | 52
☐ No

3.2 If yes, which methods?

……………………………………………………………………… | 53-54
……………………………………………………………………… | 55-56

3.4 Was this pregnancy planned?

☐ Yes | 57
☐ No

3.5 Did you attend antenatal care clinics? *If no, go to 3.11*

☐ Yes | 1
☐ No

3.6 If yes, where?

- [ ] Public
- [ ] Private

[ ] 2

3.7 How many weeks were you pregnant when you visited the clinic for the first time?

……………………………….

[ ][ ] 3-4

3.8 How many Antenatal care clinic visits did you attend?

………………………….

[ ][ ] 5-6

3.9 What medication and/or supplements did you use during pregnancy?

[ ]	Iron (Ferrous sulphate)	[ ] 7
[ ]	Folic Acid	[ ] 8
[ ]	Calcium Glutonate	[ ] 9
[ ]	Micronutrients	[ ] 10
[ ]	Enriched porridge or other nutrition supplements	[ ] 11
[ ]	ART	[ ] 12
[ ]	Hypertension	[ ] 13
[ ]	Diabetes	[ ] 14
[ ]	Other (Specify)……………	[ ] 15

3.10 If on ART treatment, did you know your status before you fell pregnant?

- [ ] Yes *If not on ART treatment…go to 3.11*
- [ ] No

[ ] 16

3.11 Did you receive breastfeeding education during your antenatal care visits?

- [ ] Yes
- [ ] No

[ ] 17

3.12 If yes, from whom?

[ ]	Professional nurse	[ ] 18
[ ]	Dietitian	[ ] 19
[ ]	Community Health worker	[ ] 20
[ ]	Other (Specify)……………………………………	[ ] 21

3.13 In the past 4 weeks or 30 days, was there ever no food to eat of any kind in your house because of lack of resource to get food?

[ ]	No *If no, go to 3.15*	[ ] 22
[ ]	Yes	[ ] 23

3.14 How often did this happen in the past 4 weeks or 30 days?

…………………… times

[ ][ ] 24-25

3.15 In the past 4 weeks or 30 days, did you or any household member go to sleep at night hungry because there was not enough food?

[ ]	No *If no, go to 3.17*	[ ] 26
[ ]	Yes	[ ] 27

3.16 How often did this happen in the past 4 weeks or 30 days?

…………………………….. times | 28-29

3.17 In the past 4 weeks or 30 days, did you or any household member go a whole day and night without eating anything at all because there was not enough food?

- ☐ No *If no, go to 3.19* | 30
- ☐ Yes | 31

3.18 How often did this happen in the past 4 weeks or 30 days?

……………………………… times | 32-33

3.19 Has this child been registered for child support grant

- ☐ No | 34
- ☐ Yes | 35

3.20 If not, what are the reasons?

……………………………………………………………… | 36-37

………………………………………………………………. | 38-39

3.21 Did you smoke cigarettes or use snuff your pregnancy?

- ☐ Yes | 40
- ☐ No | 41

3.22 Did you smoke other recreational drugs during your pregnancy?

- ☐ Opium | 42
- ☐ LSD | 43
- ☐ Barbiturates | 44
- ☐ Amphetamines | 45
- ☐ Ecstasy | 46
- ☐ Cocaine | 47
- ☐ Heroin | 48
- ☐ Cannabis | 49
- ☐ Other…………………………………….. | 50

3.23 Did you drink any alcohol during your pregnancy?

- ☐ Yes | 51
- ☐ No | 52

Mother Baby Friendly Practices at maternity faciltiy

4.1 How was your baby delivered?

- [ ] Vaginal — 1
- [ ] Ceaser — 2
- [ ] Assisted delivery — 3

4.2 Did the facility allow you to bring a companion with you?

- [ ] Yes — 4
- [ ] No — 5

4.3 Were you offered anything to eat or drink while you were in labour?

- [ ] Yes — 6
- [ ] No — 7

4.4 How soon after birth did you hold your baby?

- [ ] < 1 hour — 8
- [ ] > 1 hour — 9

4.5 Were you offered help with breastfeeding within 6 hours after birth?

- [ ] Yes — 10
- [ ] No — 11

4.6 Did you practice skin to skin contact? If yes, for how long?

- [ ] < 1 hour — 12
- [ ] 1 hour — 13
- [ ] > 1 hour — 14
- [ ] Did not practice skin to skin — 15

Exclusive Breastfeeding information

5.1 What did your baby drink or eat in the past 24 hours

- [ ] Exclusive breastfeeding — 16
- [ ] Mix Feeding — 17
- [ ] Exclusive formula feeding — 18
- [ ] Solid foods (e.g. porridge, yogurt) — 19

5.2 If mix feeding, what was given?

- [ ] Formula — 20
- [ ] Water — 21
- [ ] Porridge — 22
- [ ] Other, please specify…………………………………………… — 23

5.3 If mix feeding, please give reasons?

……………………………………………………………………………………… 24-25
……………………………………………………………………………………… 26-27
……………………………………………………………………………………… 28-29
……………………………………………………………………………………… 30-31
……………………………………………………………………………………… 32-33
……………………………………………………………………………………… 34-35

Road to Health Booklet

6.1 Is the RtHB completed correctly

Page 2 recording sheet, according to IMCI guidelines

☐ Yes
☐ No

☐ 36

Page 4 details of child and family

☐ Yes
☐ No

☐ 37

Page 5 immunizations

☐ Yes
☐ No

☐ 38

Page 5 Head circumference

☐ Yes
☐ No

☐ 39

Page 6 Neonatal info

☐ Yes
☐ No

☐ 40

Page 7 PMTCT info

☐ Yes
☐ No

☐ 41

Page 14 Birth weight plotted

☐ Yes
☐ No

☐ 42

Page 17 Birth length plotted

☐ Yes
☐ No

☐ 43

6.2 What was the **birth** weight of the baby?

_________________ g

☐☐,☐☐ 44-47

6.3 What was the **birth** length of the baby?

_________________ cm

☐☐,☐☐ 48-51

6.4 What was the baby's head circumference at **birth**?

_________________ cm

☐☐,☐☐ 52-55

Anthropometry of mother and baby

7.1 **Mother**

Weight (kg) ………………………………..

Height (cm) ……………………………….

☐☐☐,☐ 56- 60
☐☐☐,☐ 61-65

MUAC (cm) 66-68

Waist circumference (cm) 69-71

7.2 **Baby (birth - 6 weeks)**

Weight (g) 72-75

Length (cm) 76-79

Head circumference (cm) 80-83
